# The Effect of Fatty Acids and BSA Purity on Synthesis and Properties of Fluorescent Gold Nanoclusters

**DOI:** 10.3390/nano10020343

**Published:** 2020-02-17

**Authors:** Pavlína Andrýsková, Karolína Machalová Šišková, Šárka Michetschlägerová, Klára Jiráková, Martin Kubala, Daniel Jirák

**Affiliations:** 1 Department of Biophysics, Faculty of Science, Palacký University Olomouc, 17. Listopadu 12, 771 46 Olomouc, Czech Republic; pavlina.andryskova@upol.cz; 2 Department of Science and Research, Faculty of Health Studies, Technical University of Liberec, Studentska 1402/2, 461 17 Liberec, Czech Republic; sarka.michetschlagerova@tul.cz (Š.M.); klara.jirakova@tul.cz (K.J.); daniel.jirak@ikem.cz (D.J.); 3 Department of Experimental Physics, Faculty of Science, Palacký University Olomouc, 17. Listopadu 12, 771 46 Olomouc, Czech Republic; martin.kubala@upol.cz; 4 Institute for Clinical and Experimental Medicine, Videnska 9, 140 21 Prague, Czech Republic

**Keywords:** Au nanocluster, fatty acid, palmitic acid, serum albumin, cell viability

## Abstract

Fluorescent gold nanoclusters (AuNCs) are envisaged as a novel type of fluorophores. This work reports on the first comparative study investigating the effect of presence/absence/abundance of fatty acids (namely palmitic acid, PA) or other substances (like glycoproteins and globulins) in the protein (bovine serum albumin, BSA) on synthesis and properties of the final AuNCs. The most popular template (BSA) and microwave (MW)-assisted synthesis of AuNCs have been intentionally chosen. Our results clearly demonstrate that the fluorescent characteristics (i.e., fluorescence lifetime and quantum yield) are affected by the fatty acids and/or other substances. Importantly, the as-prepared AuNCs are biocompatible, as determined by Alamar Blue assay performed on Hep G2 cell line.

## 1. Introduction

Gold nanoclusters (AuNCs), an intensively studied class of fluorophores with several benefits, including high stability, extremely long fluorescent lifetime, large Stokes shift, red emission, and biocompatibility, have been examined and developed in recent years [1,2,3,4]. Due to special features, protein-encapsulated AuNCs, a promising material for fluorescence labeling in biomedical imaging [5,6,7] and sensing [1,8], have been studied to understand the role of a template to AuNCs bio-functionality [4,9].

Bovine serum albumin (BSA) serves as a frequently exploited template protein in AuNCs synthesis. It is the major plasma protein that contains 583 amino acids (MW ≈ 65,5 kDa) arranged into three domains (I–III), which split to two subdomains (A and B). Each domain is composed of 10 α-helix chains, which determine the secondary structure of the whole protein. BSA possesses 17 disulphide bridges and one free –SH group, which is responsible for the formation of a covalently linked dimer [10]. The BSA conformation is changed according to the actual pH value of the surrounding medium, hence the structures of five pH-affected forms (Extend–Fast–Normal–Basic–Aged). Several important pH values, where conformational changes are observed, can be found at 2.7, 4.3, 8, and 10 [8,11,12]. According to the recent paper [13], only BSA in the Aged form (pH > 10) interacting with Au ions forms the red fluorescent species (i.e., multiple chromophores such as Au-complexes and AuNCs, which are present on different binding sites of BSA).

The main purpose of BSA is to transport many ligands in blood, especially drugs (warfarin, L-Dopa, etc.), fatty acids (FAs), or inorganic ions (Ca^2+^, Zn^2+^, Cu^2+^, Pt^2+^, Au^3+^, etc.) [10,14,15]. Indeed, BSA is often used in research for its high similarity with human serum albumin (HSA) (approximately 76%) [16,17].

From the conformational point of view, even the binding of FAs to BSA can cause structural changes, which correspond to a structure between normal and basic forms [10,18]. Seven binding sites (FA1–7) for long-chain FA in albumins (BSA or HSA) structure have been reported in the literature [14,18,19,20,21]. These sites are distributed throughout the protein in an asymmetric way. According to NMR studies [22], high-affinity FA2, FA4, and FA5 sites are located between domains IA and IIA (FA2), in domain IIIA (FA4), and IIIB (FA5). The intermediate binding FA1, FA3, FA6, and FA7 (approximately five times weaker bond than high-affinity sites) are computed in domain IB (FA1), IIIA (FA3), on the interface between IIA and IIB (FA6), and in domain IIA (FA7) [23,24]. The binding sites generally consist of preformed hydrophobic cavities, which appear to expand upon FAs binding as the result main chain and sidechain adjustments [10]. Perhaps the most impressive example of this occurs in FA1 in subdomain IB, where a pair of stacked tyrosine residues (Tyr138 and Tyr161), which partially fill the pocket in the defatted albumin, rotate about 90° to allow them a contact with a FA tail. [10]

The molar ratio of long-chain FAs, namely palmitic acid (PA), to albumin is 0.1–2 under physiological conditions. This molar ratio is increased to the value of 6 in extreme situations (e.g., obesity or diabetes). Therefore, significant differences between fatted (i.e., BSA containing FAs) and defatted (i.e., FA free) albumin, such as the conformational, structural, thermal changes, and chemical stability, are associated with the presence/absence of FAs [10,20,25,26,27].

Oleszko et al. [25] studied the changes of BSA secondary structure after PA binding by using infrared (IR) spectroscopy and compared their results with molecular simulations performed for FA-HSA [14,20]. The IR spectroscopic results [25] demonstrated that BSA secondary structure was sensitive to the amount of bound PA when binding into high-affinity sites (whereas filling the other sites with lower affinity to FAs led to no substantial changes of protein structure). No significant differences were observed between commercially available fatted and defatted BSA for a low amount of PA addition: In both cases, an increase of α-helices content was observed [25]. In all the experiments, fatted BSA showed slightly higher (although less than 1%) content of α-helices than the defatted protein. The maximum content of α-helices corresponded to the physiological PA:BSA molar ratio. [25] 

The influence of FAs presence/absence is the most evident in the thermal stability of BSA [26,28]. According to differential scanning calorimetry (DSC) [26], fatted BSA shows a single endotherm peak (located near 69 °C) in DSC curve, with the enthalpy change of about 1000 kJ/mol. On the contrary, defatted BSA melting is reached at two different temperatures: About 56 and 69 °C, with the enthalpy changes of about 300 and 200 kJ/mol, respectively. In comparison to a conventional heating, the usage of low microwave (MW) power seems to be gentler to BSA at higher temperature [28].

Leggio et al. demonstrated the role of FAs as strong stabilizing agents in urea-induced denaturation processes [27]. Based on their results for defatted HSA and PA:HSA complexes [27], it was revealed that a multistep unfolding process took place in the case of defatted HSA; while a single step mechanism occurred in a completely fatted form of BSA.

Concerning metal cations, four binding sites are described in albumins: N-terminal site (NTS, or “Asp fragment”), vicinity of Cys34 (the only cysteine residue in BSA that does not form disulfidic bridges), multi-metal binding site (MBS or site A) at the interface of domains I and II, and site B [15]. Two of these sites, NTS and Cys34, were examined as possible binding or nucleation sites of gold (0 or III) [13]. Based on intentional cysteine residues blocking, it was evidenced [13,15] that all cysteine residues, not specially Cys34, served as the best binding sites for gold ions in albumin.

The origin, location, and influence of AuNCs on BSA have been reported [2,12,29,30]. Russell et al. [12] computed the probable center of NCs formation in domain IIB. The author observed the effect of NCs on physico-chemical properties of albumin (density, viscosity, etc.), as well as on the ability of drugs binding in the albumin-NCs system [29,30,31]. The changes in the secondary and tertiary structures of BSA due to AuNCs presence were confirmed both experimentally and theoretically [1,2,8,12].

The choice of a template type plays a critical role in the AuNCs chemistry. The effect of template sizes and amino acid contents on the final AuNCs has already been described [9]. Furthermore, the recently published paper [32] reports higher fluorescence intensity of rigidified structures (Au_10_(SG)_10_ in toluene) in comparison to un-rigidified ones (in water). The authors explain it by isolation and protection of AuNCs from the solvent environment due to aliphatic chains of a phase-transfer agent (i.e., a molecule causing the phase-transfer from aqueous to organic phase) and induced restrictions of molecular vibrations or rotations of ligands surrounding NCs. Therefore, our work is devoted to the examination of FAs absence/presence/abundance in BSA on AuNCs formation and fluorescent properties. We have intentionally chosen BSA for several reasons: (i) It is the most frequently used and studied protein for AuNCs synthesis; (ii) it is an important transport protein of species in blood, enabling their circulation in body, which has consequences for NCs application in sensing and imaging; and (iii) it is also known as a resilient protein (e.g., it can even refold into its natural conformation after some harsh processes of denaturation). Indeed, we assume that the well-known seven binding sites of fatty acids, as determined in [10], and several possible locations of AuNCs (of different sizes), determined e.g., in Russell et al. [12], within albumin structure can mutually influence, thus leading to changes in AuNCs fluorescence features.. The next aim of this study was the investigation of the effect of different BSA purities (from the chemical point of view) on AuNCs formation and fluorescent features because different sources of BSA are employed by researchers. Therefore, the commercially available 98% BSA (further denoted as 98BSA) and 98% BSA fatty acid free (denoted as df98BSA) were used for AuNCs synthesis (98BSA-AuNCs and df98BSA-AuNCs, respectively). Moreover, the addition of PA into the df98BSA and the effect of BSA purity (using 96% BSA, denoted as 96BSA) on AuNCs formation and the fluorescent properties were examined. The as-prepared materials were physically and chemically characterized by UV-Vis absorption, fluorescence (steady-state and time-resolved), circular dichroism, dynamic light scattering, zeta potential measurements, and infrared absorption spectroscopy. Furthermore, to test the biocompatibility of AuNCs, the viability tests were performed with Hep G2 cell line for selected samples.

## 2. Materials and Methods 

*Chemicals:* Various purity (≥96%, ≥98.0%, and ≥98.0% fatty acids free) of bovine serum albumin, gold(III) chloride trihydrate (HAuCl_4_·3H_2_O), sodium hydroxide (NaOH), and palmitic acid (PA) were purchased from Sigma-Aldrich (St. Louis, MO, USA) and used for all experiments without any further purification. Deoxygenated deionized (DO/DI) water prepared by purging Milli-Q purified water (Millipore Corp., Bedford, MA, USA) with N_2_ for 30 min was used in all the experiments including material synthesis.

*Synthesis of gold nanoclusters*: Gold nanoclusters AuNCs were prepared by the reduction of HAuCl_4_ using BSA of different purity: 96% BSA (96BSA), 98% BSA (98BSA), and fatty-acids-free BSA (df98BSA). In all three cases, BSA served as the reducing and simultaneously stabilizing agent; the whole reduction was performed in an alkaline environment (pH = 11). In a typical procedure, 1 mL of BSA (40 mg/mL) and 1 mL of HAuCl_4_ (10 mM) were mixed under vigorous stirring. Ninety seconds later, NaOH (0.2 mL, 1 M) was added and stirred for the next 90 s. Then, the yellow solution was exposed to microwave heating (in a standard kitchen microwave) for 10 s using the power of 150 W. Microwave irradiation supports the reduction process [1,8,33]. After 20 min of aging at room temperature, the final nanoclusters (96BSA-AuNC, 98BSA-AuNCs, df98BSA-AuNCs) were characterized and then stored at 4 °C (fridge) for later use.

*BSA reference samples*: (96BSA-ref, 98BSA-ref, df98BSA-ref) were prepared by the same experimental procedure with the only exception that the addition of HAuCl_4_ was omitted.

*Influence of palmitic acid addition*: Solutions of palmitic acid (15 mg/mL in ethanol) were mixed with df98BSA (40 mg/mL) in the PA:BSA molar ratios of 0.1, 0.5, 1, 2, and 6. These PA:df98BSA were used for MW-assisted NC synthesis.

Moreover, the solution of palmitic acid (15 mg/mL in ethanol) was also introduced into the samples of df98BSA-AuNC immediately after their synthesis in PA:df98BSA molar ratio of 6. Physico-chemical properties of these samples were characterized four days after their synthesis.

*Model of PA:HSA-AuNCs* was prepared by using open-source PyMOL 1.3. molecular graphics system (http://www.pymol.org). The HSA complexed with PA were loaded as 1e7h structure from PBD [34]. The cysteine (cyan), tyrosine (pink), and tryptophan (blue) residues and PA (red chains) were highlighted in the HSA structure. The possible locations of AuNCs (yellow globules) were visualized according to the literature [12].

*Methods of characterization*: Fluorescence of NCs was recorded on JASCO F8500 (Jasco, Tokyo, Japan) using 1 cm quartz cuvette. Excitation at 280 nm was used for recoding emission spectra in the range of 300–850 nm. Data were recorded with the interval of 0.1 nm and scan speed of 500 nm/min. The spectral correction was used. Emission–excitation 3D maps were measured in the following ranges: Excitation 250–420 nm and emission 300–800 nm. Data were recorded with the interval of 5 nm and scan speed of 5000 nm/min. Measurements were performed at RT and the protein concentration was adjusted to 1 mg/mL by diluting with deionized water.

*Fluorescence decays* of AuNCs were measured on a TCSPC fluorometer PicoHarp300 (PicoQuant, Berlin, Germany), using pulsed laser diode centered at 445 nm for the excitation, with the repetition frequency of 2.5 MHz (hardware lower limit). Data were recorded into a histogram on the time scale of 0-400 ns, where the time-width of one channel was 128 ps. The instrument response function (IRF) was obtained using distilled water as a scatter, and estimated FWHM(IRF) was 0.512 ns. Emission was detected under magic-angle conditions at 640 nm, and the emission bandpass was 16 nm in all cases. The data were acquired at 295 K for 20 min or until 20,000 counts in the peak-channel were achieved. Fluorescence decays were fitted using the FluoFit 4.2.1 software (PicoQuant, Berlin, Germany) as a sum of exponentials:

(1) $$I\left(t\right)=IRF⊗\overset{n}{\underset{i=1}{\sum }}A_{i}e^{-\frac{t}{\tau_{i}}}$$

Because the lifetime of the longest decay component was comparable to the period of excitation pulses, cyclic excitation was considered during data fitting. The quality of each fit was evaluated by the χ^2^_R_ and distribution of residuals. The τ_i_’s 68% confidence intervals (corresponding to ±1 SD in Gaussian distribution) were estimated using support plane analysis. 

*The quantum yield (QY*) was measured using tryptophan (QY = 0.12 [35]) as a reference. The QY can be calculated by the following equation:

(2) $$QY=QY_{r}*\frac{I}{I_{r}}*\frac{A_{r}}{A}*\frac{n^{2}}{n_{r}^{2}}$$

where *I* is integral of fluorescence intensity, *A* is the absorbance, *n* is the refractive index, and *r* subscript indicates the reference (similarly as in [36]).

*Changes in the protein structure* were evaluated by ATR-IR Nicolet (Nicolet, Madison, WI, USA) with diamond crystal at the range of 4000–400 cm^−1^ and the resolution of 4 cm^−1^. Then, 2 µl of a sample were dried on the diamond crystal on air at RT for 10 min. Spectra were baseline corrected in Omnic software. Deconvolution of amide I band was performed in Origin software (Nicolet, Madison, WI, USA) using four gaussian curves (fit quality described by R^2^ value of 0.9999 or better was achieved). 

*The secondary structure* of the protein was determined by a circular dichroism (CD) JASCO J-815 (Jasco, Tokyo, Japan) in 0.1 cm quartz cell. CD spectra were taken in the range of 190–400 nm and each spectrum was the average of four scans. The secondary structure was computed by algorithm BeStSel (Beta Structure Selection). The algorithm carried out a detailed secondary structure analysis providing information on eight secondary structure components [37,38].

The content of α helix was also calculated using molecular residue ellipticity (MRE) with OriginPro 2017 (OriginLab, Northampton, MA, USA). The content of α helix was estimated based on Equations (3) and (4) (similarly as in [11,39]),

(3) $$MRE=\frac{\Theta }{10*cln}$$

(4) $$\alpha -helix=\frac{-MRE\left(208\right)-4000}{33000-4000}$$

where *Θ* is ellipticity derived from CD in deg·cm^2^·mol^−1^, *c* the molar concentration of the protein (in our case 3.8 µM), *l* is the length path of the cell (in our case 0.1 cm), *n* is the number of amino acids residues (in our case 583), and MRE determined from CD spectrum at the wavelength of 208 nm.

*Hydrodynamic size and zeta potential* of NCs were characterized by dynamic light scattering with Zetasizer Nano ZS (Malvern Instruments Ltd, Malvern, UK) equipped with a He-Ne laser (633 nm), at 25 ± 1 °C. Measurements of hydrodynamic radius of systems were performed at the protein concentration adjusted to 1 mg/mL by diluting with deionized water. Zeta potential was recorded for samples without dilution.

Several times we attempted to measure our samples by using ESI MS (electrospray ionization mass spectrometry), but the resulting spectra were very noisy, hence no relevant information was obtained. Similarly, MALDI MS (matrix-assisted laser desorption/ionization mass spectrometry) spectra recording was also unsuccessful. MS analysis of protein-templated gold nanoclusters usually requires a higher concentration of the protein samples. Although we prepared very concentrated samples, they started forming a gel, which was difficult to take by a pipette. Therefore, measurements were disabled. These extensive studies with mass spectrometry are especially difficult as reproducible and reliable mass spectra of such systems require careful optimization [40].

*Tests of cells viability:* To measure the viability of labeled cells, Alamar blue assay (Sigma Aldrich, St. Louis, MO, USA) was performed. Alamar blue assay is designed to study cytotoxicity of various agents, the quantitative measurement responds to the metabolic activity of the cells. Human hepatoma cell line HepG2 was plated in 96-well plate (20,000 cells per well) and incubated for 24 h. Cells were then labeled with various concentrations (12.5, 25, 50 µg·Au/mL) of 98BSA-AuNCs, df98BSA-AuNCs, and 96BSA-AuNCs for 24 h. Prior to spectrophotometric analysis, 10% solution of Alamar blue in cultivation medium was added to each well. After that, the plate was incubated for 4 h at 37 °C. The cell viability was then assessed by measuring the absorbance at wavelengths of 570 nm and 600 nm using reader Tecan The Infinite 200 PRO (Tecan Group Ltd., Männedorf, Switzerland). Viability for treated samples was determined as a percentage of control (viability of control was set as 100%).The experiment was performed in triplicate and repeated three times.

## 3. Results and Discussion

**Figure 1:**
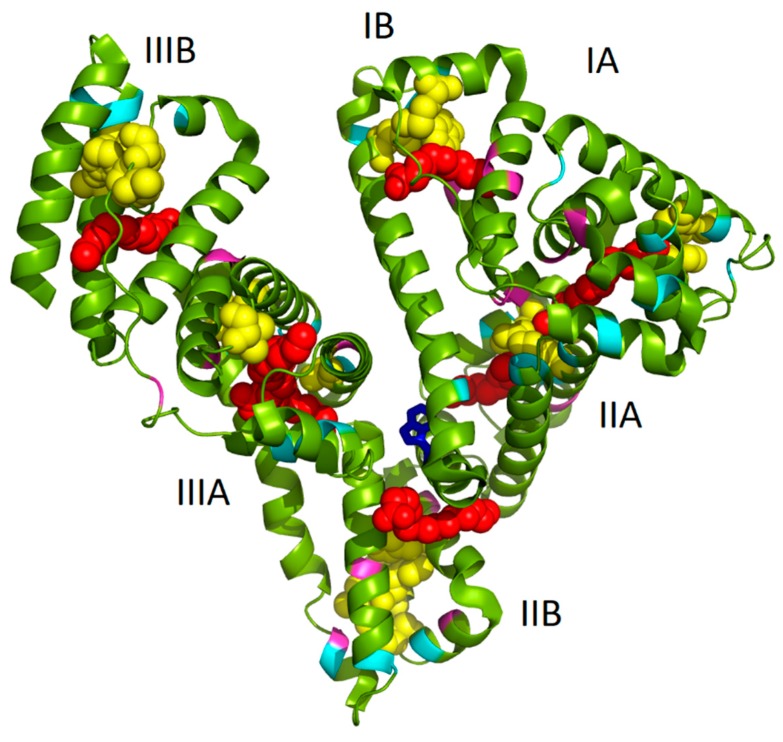
Palmitic acid (PA):albumin-gold nanoclusters (AuNCs) model, adapted from PA:HSA structure (1e7h in PDB, [34]), shows seven binding sites for palmitic acids (PA) and possible locations of AuNCs proposed by Russell et al. [12]. The PA (red chains) and AuNCs (yellow globules) are visualized in the protein together with highlight of cysteine (cyan), tyrosine (pink), and tryptophan (blue) residues.

As it has been already mentioned in the Introduction, we assume that the well-known seven binding sites of FAs and several possible locations of AuNCs (of different sizes) within albumin structure can mutually influence and thus lead to changes in AuNCs fluorescence features. In Figure 1, the structure of HSA complexed with PA molecules (1e7h from PDB, [34]) fused together with AuNCs positioning (determined by Russell et al. [12]) is depicted. It should be noted that the structure of HSA with PAs was exploited due to the structural similarity of HSA and BSA and because no BSA structure with PAs was available in the protein database. Evidently, several PAs were located very close to AuNCs binding sites (Figure 1), thus changing the polarity of AuNCs micro-environment. This could mimic the situation of NCs phase transfer from aqueous to nonpolar solvent, accompanied by an increase of fluorescence as published recently in [32]. Therefore, in this paper, the effect of fatty acids absence/presence/abundance in the protein on the final AuNCs and Au-BSA complexes formation, as well as their physico-chemical properties, were investigated. 

Later in the work, we used (in the next text, we are using) only the abbreviation AuNCs, but we are aware of the fact that not only nanoclusters (where just Au–Au bonds can be encountered), but also Au(I)-BSA complexes (containing namely Au–S bonds) were present within the samples due to several possible binding sites of Au(III) on BSA as it has been discussed many times in recent literature [13,32]. According to the specific conditions of the selected synthesis, either a partial reduction of Au(III) to Au(I) [32], or reduction to Au(0) and Au(I) have been evidenced [41]. 

In the following section, structural changes of BSA in the course of the selected AuNCs synthetic procedure are evaluated for the case of the natural (98BSA) or fatty acid free (df98BSA) proteins used in this study. Then, step-by-step changes in BSA hydrodynamic diameter accompanying the process of AuNCs formation are listed and discussed together with zeta potential values of the final AuNCs. Subsequently, fluorescent features of the final AuNCs prepared in the presence of these two types of templates can be mutually compared and discussed. Finally, the effect of addition of increasing PA concentration, either directly to df98BSA before the process of AuNCs formation, or to the final AuNCs prepared by using df98BSA, can be revealed.

BSA purity (from the chemical point of view), meaning the comparison between 98BSA and 96BSA used as templates, may be also important for the final AuNCs features. Therefore, this aspect was considered, and the results are discussed in Section 3.2.

From the practical viewpoint, the stability of fluorescent properties and biological tolerance (cell viability) of the final AuNCs were determined and discussed in detail in Section 3.3 and Section 3.4, respectively.

### 3.1. Effect of Fatty Acids Absence/Presence/Abundance on BSA-AuNCs Formation and Fluorescent Characteristics

#### 3.1.1. BSA Structural Changes in the Course of AuNCs Synthesis

**Scheme 1:**
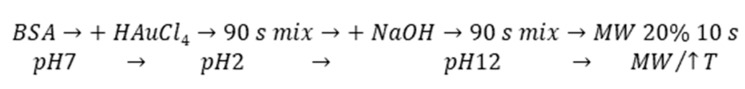
The key steps of microwave (MW)-assisted AuNCs synthesis: Namely, pH changes and MW treatment.

The MW-assisted synthesis was selected as a rapid, effective, and gentle method for our final AuNCs preparation using BSA. According to Scheme 1, the one pot synthesis of AuNCs can be divided into key steps, which include extreme pH changes and MW irradiation (non-thermal effect) with uniform heating. These special conditions significantly affected template structure, which was crucial for protein unfolding and opening of amino acid residues to metal (Au(III)) binding (Cys) and reduction (Tyr). Although the exact position within albumin structure where the gold complexation starts remains unrevealed, even after experiments exploiting synchrotron [32], there is a generally accepted scenario: Cysteine residues are responsible for the first interaction with Au(III), followed by Au–S covalent bond formation within the first hour of the synthesis. In the next few hours (0–3 h), disulfide bridges present in the protein are cleaved and, simultaneously, the natural conformation of the protein lost. During the next several hours (3–12 h), AuNCs become more ordered inside the protein due to structural optimization [32]. Furthermore, it is known that tyrosine and tryptophan are able to reduce Au(III) under strongly alkaline pH values to Au(0), thus forming AuNCs and nanoparticles [42,43,44]. Therefore, cysteine, tyrosine, and tryptophan residues are visualized in Figure 1. In several cases, their position was in the close vicinity of AuNCs or FAs, interacting with them most probably through electrostatic and coordination covalent bonds or hydrophobic interactions, respectively. It should be noted that the structure in Figure 1 is a static image of the albumin molecule and does not reflect the dynamics of the process of AuNCs formation. The conformational changes of the protein were thus investigated for our cases of different types of BSA used.

BSA possess mostly α-helical structure, therefore the relative changes in BSA structure in the course of AuNCs synthesis can be evaluated based on alpha helix content determined by CD—Figure S1 (Supporting Information). In general, one positive absorption band at 190 nm (π→π*) and two negative peaks at 208 nm (π→π*) and 222 nm (n→π*) were characteristic for α-helix. Figure S1A (Supporting Information) confirms the previously discussed phenomenon of higher α-helicity in 98BSA in comparison to df98BSA [25]. In Figure S1B,C (Supporting Information), the changes of α-helix content of 98BSA and df98BSA caused by pH changes or MW treatment during our AuNCs syntheses are shown. In both cases (98BSA, as well as, df98BSA), the unfolding of the protein was observed as evidenced particularly at 208 nm: α-helicity content decreased, and simultaneously, random coil content increased.

There are several different approaches used for the evaluation of alpha helix content of a protein based on CD spectra changes. In Table 1, the results of two of them are summarized: Either the CD spectra evaluated by BeStSel algorithm (detailed structural changes shown in Table S1 (Supporting information)), or calculated values based on Equations (3) and (4) mentioned in the Experimental section (similarly as in [11,39]). Both approaches confirmed the reduction of α-helicity and simultaneous increase of other structures (like turn and random coil) in the course of AuNCs formation. Moreover, slightly higher stability and content of α-helix were determined for 98BSA and the samples derived from this BSA type in comparison to df98BSA.

Infrared spectroscopy is the second most frequently and commonly used spectroscopic technique for protein structure and dynamics studies. IR spectral regions characteristics for peptide linkage are: Amide I (1600–1690 cm^−1^), amide II (1480–1575 cm^−1^), amide III (1229–1301 cm^−1^), amide A (3300 cm^−1^), and amide B (3100 cm^−1^) [45]. The changes in amide I region and band deconvolution into several distinct peaks have been discussed in the recent literature when the secondary structure of a protein and its changes investigated [41,46,47]. While the presence of a band at 1653 cm^−1^ is attributed to the buried α-helix in the native BSA, the band at 1624 cm^−1^ is assigned to the exposed α-helix; the bands located at 1672 and 1682 cm^−1^ are characteristic for turn structures, and the band at 1648 cm^−1^ indicates the presence of random coil structures of the protein in AuNCs-BSA [41,46,47].

**Figure 2:**
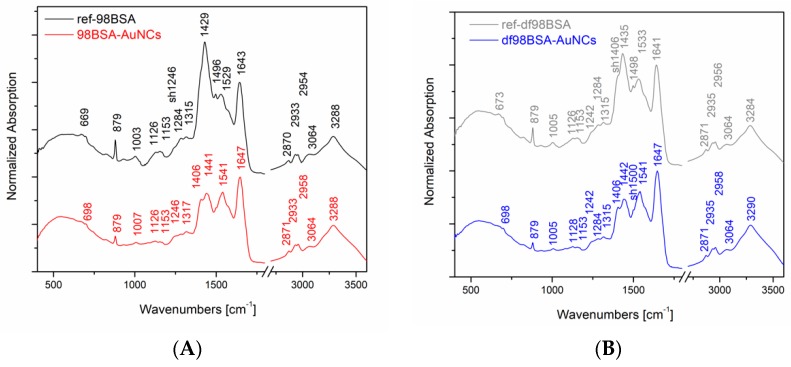
Normalized IR absorption spectra (to the band at around 1643 ± 4 cm^−1^) of AuNCs prepared by using either (**A**) 98BSA or (**B**) df98BSA. Reference IR spectra of the same type of BSA treated by the same manner as that applied in the AuNCs synthesis, however without the addition of HAuCl_4_, are shown for the sake of a direct comparison.

In our experiments, we performed IR absorption measurements of the final AuNCs and the appropriate references, as it is shown in Figure 2. For the sake of a direct comparison, the spectra were normalized with respect to the band of a maximal intensity in amide I region (i.e., 1643 ± 4 cm^−1^). Systematically, there was a shift of this band maximum toward higher wavenumbers in the IR spectra of the final AuNCs in comparison to their references. Considering the abovementioned, and in the literature [41], discussed spectral deconvolutions of amide I region, we performed the deconvolution of this region as well in order to determine the percentage of buried and exposed alpha helix, random coil, and turn structures. The results of curve fitting using gaussian curves are presented in Table S2 (Supporting Information). Obviously, the content of alpha helices, either buried or exposed, decreased in both samples (98BSA, as well as, df98BSA) containing AuNCs in comparison to their references—Table S2 (Supporting Information). Simultaneously, the percentage of random coil increased. The increase of structural disorder was more pronounced in df98BSA-AuNCs, which was also consistent with the results derived from CD spectroscopy.

Furthermore, comparing the IR spectra of AuNCs and their appropriate references, substantial differences in amide II region were detected as well—Figure 2. Since vibrational signals in amide II region reflect CN stretching and NH bending, the observed differences further support the changes of secondary structure of BSA due to the synthetic procedure of AuNCs.

Moreover, significant changes in the IR absorption intensity of the peak located at 879 cm^−1^ are obvious in Figure 2: A decrease in intensity when going from the reference to the AuNCs sample (both cases: 98BSA and df98BSA). This sharp peak could be attributed to tyrosine residues, which are partially (not all of them) oxidized during AuNCs synthesis and therefore their intensity in IR spectra decreases.

#### 3.1.2. Size Changes in the Course of AuNCs Synthesis and Zeta Potentials of the Final BSA-AuNCs

To follow the changes of the protein size during AuNCs synthesis, dynamic light scattering (DLS) technique was intentionally chosen as it allows the estimation of sizes directly in solutions, i.e., hydrodynamic diameter of dispersed particles in their medium. Especially, the relative size changes in the course of particular AuNCs formation evaluated as the difference between the initial and final values are important. Other techniques, such as HR-TEM (high-resolution transmission electron microscopy) and/or MS (mass spectroscopy; either matrix-assisted laser desorption ionization, MALDI, or electrospray ionization, ESI), frequently used by researchers, are more accurate in some view; however, they require the samples to be dried and/or ionized to gas phase, respectively. Consequently, the samples are measured in an unnatural state, which could cause severe discrepancies among data. 

Owing to the at least partial unfolding of BSA during the process of AuNCs formation (as evidenced by CD and IR spectroscopy and discussed in terms of conformational changes), hydrodynamic diameters determined by DLS increased during the formation of BSA-AuNCs as it can be seen in Table S3 (Supporting Information). Data of the hydrodynamic diameter determined by DLS for the initial proteins are listed in Table S3 (Supporting Information) as well, in order to (i) validate our measurements and (ii) demonstrate that we are dealing with monomer protein molecules in the beginning. The size of albumin was influenced by the presence of FA in the system (compare df98BSA and 98BSA values: 8.6 ± 0.5 vs. 6.4 ± 0.2)—Table S3 (Supporting Information). Indeed, the hydrodynamic radius of 98BSA was higher than df98BSA by approximately 2 nm due to an adaptation of the main chain and side chain upon FA binding as already discussed in the literature [10]. Our DLS measurements were thus validated and can be further employed for size changes determination during the process of AuNCs synthesis using different types of BSA and followed step-by-step.

In spite of the fact that (since) many researchers use the synthetic procedure described in Xie et al. [2], i.e., without MW irradiation, we decided to evaluate the changes in diameter in two steps: (i) After the alkalization—denoted as Δ1 in Table S3 (Supporting Information), and (ii) after MW treatment—labelled as Δ2 in Table S3 (Supporting Information). Obviously, Δ1 (being 2.4 nm and 6.8 nm for samples 98BSA-AuNCs and df98BSA-AuNCs, respectively) exceed the experimental error far more (being of 1.4–3.5 nm). On the contrary, Δ2 values (3.6 nm for 98BSA-AuNCs, 1.2 nm for df98BSA-AuNCs) are almost negligible for df98BSA taking into account the experimental error. There are at least three processes that may occur and lead to the changes of protein diameter in the course of BSA-AuNCs formation: (a) BSA unfolding—until 2.5 nm changes in diameter (according to the literature [48,49]), (b) NCs formation—changes for one AuNC max. 1.4 nm or less [48], and (c) dimer formation. Considering Δ1 and Δ2 values (being less than 7 nm), only (a) and (b) processes can be encountered in the course of BSA-AuNCs synthesis. As revealed by our CD results (presented in Table S2 (Supporting Information)), a significant decrease of alpha helix content was observed, hence process (a) can be the major contribution in Δ1; in other words, BSA unfolding dominated after alkalization. On the other hand, AuNCs formation was more pronounced during the next steps. The highest Δ1 value achieved in df98BSA system corroborated the lack of protein structure stabilization due to the absence of FAs; while the highest Δ2 value encountered in 98BSA confirmed multiple AuNCs formation (which is consistent with increased fluorescence and QY as it will be shown in the next section).

The zeta potential values of the AuNCs were around −14.5 mV for df98BSA-AuNCs and −16.0 mV for 98BSA-AuNCs, thus a relatively good long-term stability of AuNCs can be assumed.

#### 3.1.3. Absorption and Fluorescence of the As-Prepared AuNCs 

Figure S2 (Supporting Information) displays the UV/Visible absorption and emission spectra of 98BSA-AuNCs. The absorption spectrum of AuNCs showed a significant peak with the maximum at 280 nm, stemming from aromatic amino acids (namely Trp, Tyr) in albumin, and a long tail reaching the visible region (consequently, the samples are orange in color). The absence of any surface plasmon resonance peak (SPR, extinction around 500–600 nm for spherical gold nanoparticles) indicated no gold nanoparticles’ formation in freshly prepared and few-days aged samples. On the contrary, several months (three and more)-aged samples manifested themselves by a characteristic SPR peak if stored in the dark under ambient conditions (note: Our samples were intentionally not dialyzed, thus a further growth of some of multiple AuNCs was enabled). 

The fluorescence broad emission band (FWHM 105 nm) of the as-prepared AuNCs with the maximum at 650 nm was obtained for the excitation at 280 nm (Figure S2 (Supporting Information)). The Stokes shift that exceeded 300 nm of fluorescent AuNCs was substantially larger compared to conventional organic fluorescent probes. 

**Figure 3:**
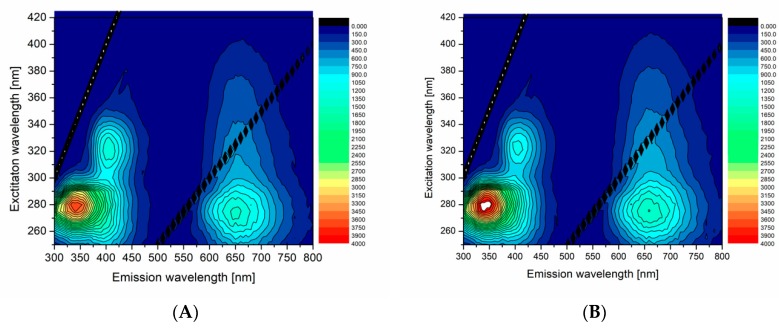
3D excitation-emission maps of (**A**) 98BSA-AuNCs and (**B**) df98BSA-AuNCs. Note: Strong Rayleigh scattering first- and second-order maxima are visible as straight lines in these 3D maps and represent artifacts, which do not belong to the samples.

In order to compare and demonstrate fluorescent properties of df98BSA-AuNCs vs. 98BSA-AuNCs in more detail, we present the excitation-emission 3D maps in Figure 3. The intensive 280/340 nm ex/em peak came from Trp residues in BSA and was partially quenched in AuNCs. In 98BSA-AuNCs (Figure 3A), the fluorescence emission at 340 nm was reproducibly by approximate 10% lower than in df98BSA-AuNCs (Figure 3B).

The origin of the less intensive 320/405 nm ex/em peak is often attributed either to the oxidation products of Tyr residues in BSA, or to small size blue-emissive AuNCs [33]. This peak provided evidence about the presence of multiple binding sites of AuNCs, which grew to various sizes according to the steric restrictions of their surrounding within the protein structure.

The significant ex/em peak at 280/650 nm stemming from bigger red-emissive AuNCs had a characteristic elliptical shape in the 3D map enabling the choice of a broad range of excitation wavelengths (from UV until visible). In this elliptical area, 98BSA-AuNCs emitted more intensively than df98BSA-AuNCs (contrary to the emission observed in the region around the 340 nm). Since no shift in the peak position was observed when comparing 98BSA-AuNCs and df98BSA-AuNCs—Figure S3 (Supporting Information)—(albeit the slight hydrodynamic diameter increase detected—see Table S3 (Supporting Information)), we can assume that no changes occurred in the polarity/hydrophobicity of NCs or Trp (the major component of BSA emission) micro-environments.

Quantum yield (QY) and fluorescence lifetime (τ), listed in Table 2, belonged to the most important characteristics of AuNCs. The QY of 98BSA-AuNCs and df98BSA-AuNCs were determined to be 6.2% and 5.7% (with standard deviations of 0.2%), respectively. The results clearly demonstrate that the presence of FA in albumin (stabilizing the protein structure) can lead to slightly increased QY values of AuNCs. With respect to the close vicinity of FA to AuNCs in BSA structure—see Figure 1—it can be deduced that the polarity of the AuNCs micro-environment decreased. In accordance with the literature [32], the fluorescence intensity was then increased.

The fluorescence lifetime values for AuNCs were determined by TCSPC (Table 2). Four components were required to adequately fit the data with χ^2^_R_ close to 1.00 and random distribution of residuals (Figure S4A–C (Supporting information)). The lifetime was composed of one major long lifetime component (more than 96% contribution to the steady-state intensity) and three minor short lifetime ones. The 98BSA-AuNCs manifested itself by the value of 369 ns as a major lifetime component, which was slightly shorter than that of df98BSA-AuNCs (406 ns) However, the confidence intervals were rather broad. 

According to the literature [33], the origin of a long lifetime component could be attributed to Au–Au interaction, ligand-to-metal charge transfer, thiol–Au^+^ complex formation, or triplet-singlet transition.

#### 3.1.4. Effect of Palmitic Acid Addition

Fatty acids are known to stabilize albumin structure, which then resists pH or temperature changes more easily [25,26]. As it could be seen in the previous section, there are differences in fluorescent properties (namely in QY and lifetime values) of the final AuNCs prepared by using BSA containing either natural amount (98BSA), or no FAs (df98BSA). Therefore, we assume that AuNCs characteristics can be influenced by the abundant concentration of FAs added to BSA.

**Figure 4:**
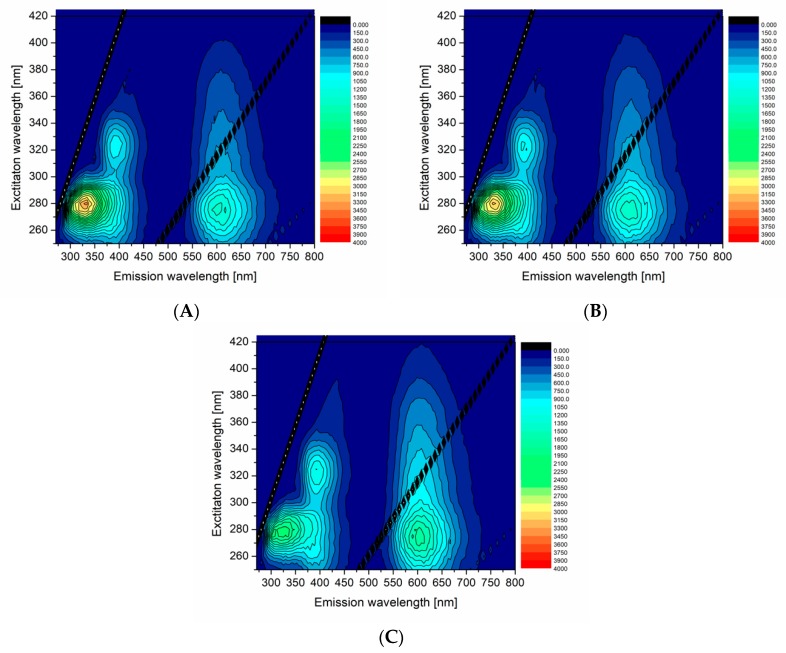
3D excitation-emission maps of PA:df98BSA-AuNCs in molar ratios: (**A**) 1:1, (**B**) 2:1, and (**C**) 6:1. Note: Strong Rayleigh scattering first- and second-order maxima are visible as straight lines in these 3D maps and represent artifacts, which do not belong to the samples.

The effect of FAs, namely palmitic acid (PA), addition was examined by fluorescence. The PA was added to df98BSA at various molar ratios (PA:BSA being 1:1, 2:1, and 6:1), which was subsequently used as a template in the above-described synthesis of AuNCs—the samples were then labelled as PA:df98BSA(molar ratio)-AuNCs. Figure 4 shows 3D excitation-emission maps of the selected PA:df98BSA(molar ratio)-AuNCs. The spectra demonstrated a reduced fluorescence intensity at ex/em 280/340 nm (attributed to Trp residues) with the increasing content of PA in dfBSA. Simultaneously, the emission maximum of AuNCs (around 650 nm) became more intensive with the PA concentration increase (compare Figure 4A with Figure 4B,C). Quantum yields of these samples, summarized in Table 3, corroborated the increasing fluorescence of AuNCs with increasing PAs content. The hypothesis about FA changing micro-environment of AuNCs was thus confirmed. 

Virtually negligible increase of the mean hydrodynamic diameter for the pre-synthetically modified PA:df98BSA-AuNCs, evaluated in Table 3, was revealed for increasing PA content. Similarly, zeta potential increased in absolute values, which is consistent with the augmenting content of carboxylic groups of Pas, thus charging the PA:df98BSA-AuNCs more negatively than df98BSA-AuNCs (−14.5 ± 1.6 nm, Table S3 (Supporting Information)). 

The changes in the secondary structure caused by PA addition into dfBSA were examined by infrared spectroscopy (Figure S5 (Supporting Information)) and revealed substantial differences in amide I and amide II regions (namely, in peak positions and their relative intensities). Similar to the structure of defatted albumin (df98BSA), 98BSA structure was affected by an abundant content of PA (see IR spectra shown in Figure S5 (Supporting Information). However, the changes in peak positions and their relative intensities were less pronounced when comparing infrared signals of PA:98BSA-AuNCs (Figure S5A (Supporting Information)) with PA:df98BSA-AuNCs (Figure S5B (Supporting Information)).

Furthermore, the effect of PA on the fluorescent features of AuNCs was investigated by adding PA to the as-prepared df98BSA-AuNCs in 6:1 molar ratio (PA:df98BSA) right after AuNCs synthesis—sample denoted as df98BSA-AuNCs:PA(1:6), i.e., the post-synthetic modification of AuNCs by PA addition was performed contrary to the pre-synthetic modification of df98BSA (i.e., prior to AuNCs synthesis). 3D excitation-emission maps of the samples of df98BSA-AuNCs, PA:df98BSA (6:1)-AuNCs, and df98BSA-AuNCs:PA (1:6) are mutually compared in Figure S6A–C (Supporting Information), respectively.

In the case of the PA addition prior to the formation of the AuNCs (Figure S6B (Supporting information)), the emission maximum of AuNCs (around 650 nm) became more intensive with the reduction of fluorescence intensity at ex/em 280/340 nm (attributed to Trp residues), as it was discussed above while commenting on Figure 4. In the case of post-synthetic modification (Figure S6C (Supporting information)), the PA addition most probably led to changes of surface polarity of BSA and micro-environment of AuNCs with an overall increase of fluorescence. Last but not least, quantum yields, presented in Table 3, manifested themselves by very similar values: Around 7% for pre-, as well as post-synthetic, modifications of df98BSA.

Taking into account the results concerning PAs addition, either pre- or post-synthetically, its positive impact on AuNCs fluorescent features was evidenced. We assume that sort of rigidifying BSA structure due to PAs addition is the main reason. PAs can interact with AuNCs by hydrophobic interactions, which mimic the phase transfer published in [32] and lead to fluorescence intensity increase. Summarizing this chapter, it can be stated that the content of PA in BSA plays a crucial role in the final AuNCs formation and has a significant impact on AuNCs fluorescent characteristics.

### 3.2. Effect of BSA Purity 

In order to investigate the effect of impurities (other substances) present in albumin on the final AuNCs formation and properties, 96BSA was used as the template and 96BSA-AuNCs were prepared, and their fluorescent features measured. The results are visualized in Figure S7 (Supporting Information) and can be directly compared with those obtained for 98BSA and 98BSA-AuNCs, respectively. 98BSA and 98BSA-AuNCs were selected for the comparison since they represented the systems with the highest α-helicity (Table 1), highest fluorescence emission intensity (at ex/em 280/650 nm) (Figure 2A), and QY (Table 2), as demonstrated throughout the previous chapter. The characteristics obtained for 96BSA-AuNCs were worse than those of 98BSA-AuNCs; namely, QY essentially decreased (Table 2).

The lifetime values of 96BSA-AuNCs, recorded by using TCSPC and presented in Table 2 (for the sake of a direct comparison with the two other albumin-AuNCs included in this study), changed; indeed, a prolongation of the lifetime longest component was revealed. Since the longest lifetime component was related to AuNCs and/or Au-protein complexes (attributed to Au–Au interaction, ligand-to-metal charge transfer, thiol–Au^+^ complex formation, or triplet-singlet transition, according to the literature [33]), this change must be caused by the presence of impurities in BSA structure. 

According to the producer description, 96BSA contains glycoproteins and globulins; therefore, more impurities (from the chemical point of view) besides fatty acids. The presence of an increased amount of impurities could probably lead to lower emission of the final 96BSA-AuNCs in comparison to 98BSA-AuNCs (compare Figure S7B (Supporting Information) and Figure 2A). This lower emission of 96BSA-AuNCs may be related to a decreased formation of AuNCs. Indeed, the formation of AuNCs could be suppressed because the residual BSA fluorescence (280/340 nm ex/em) was always higher in 96BSA-AuNCs than in 98BSA-AuNCs (compare Figure S7B (Supporting Information) and Figure 2A). This result might be caused by the content of glycoproteins and globulins, i.e., these species also reacted with Au (III) and decreased the available Au(III) concentration; consequently, the formation of AuNCs in 96BSA-AuNCs sample was decreased. 

In general, the access of auric ions to reactive amino acid residues on protein surface was an important factor in AuNCs formation, as can be deduced from IR and CD spectra, and the structure of BSA differs in 96BSA and 98BSA. Therefore, the accessibility of cysteine and tyrosine residues was probably the most different in 96BSA vs. in 98BSA, which consequently influenced the AuNCs formation and properties.

The final BSA-AuNCs approximately doubled in diameter in comparison with the initial protein—see Table S3 (Supporting Information), which was most probably induced either by multiple AuNCs content, or protein dimer formation. The latter may be the case of 96BSA.

### 3.3. Stability of AuNCs 

**Figure 5:**
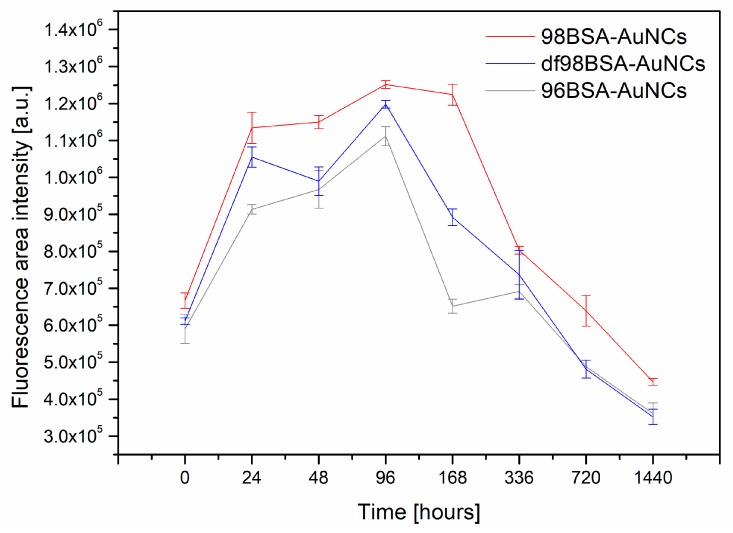
Plot of integral fluorescence intensity of 96BSA-AuNCs (grey), 98BSA-AuNCs (red), and df98BSA-AuNCs (blue) as a function of time elapsed from AuNCs synthesis.

In the literature, pH [9,46], temperature [9,33], or chemical [1,27,30] stability of AuNCs are often studied during short-term periods. In our work, the long-term stability of AuNCs was evaluated based on fluorescence intensity changes followed for several months, which is considered beneficial and more relevant for long-term in vivo applications. The data on long-term stability were published usually only for 14 days [33] or dry samples [1,2]. On the contrary, the stability of our AuNCs was investigated by using their solutions, which were stored at 4 °C in the fridge to minimize any type of degradation (particularly that related to the protein, or further NCs growth until nanoparticles, which may occur when stored at RT). The values of integral fluorescence intensity (the excitation at 280 nm and emission in the range 550–800 nm) of 96BSA-AuNCs, 98BSA-AuNCs, and df98BSA-AuNCs are plotted as a function of time (expressed in hours) in Figure 5.

Obviously, all three samples manifested themselves by very similar trends in integral fluorescence intensity changes: Increased in time until reaching maximum and then quickly decreased (basically within 7 days for 96BSA-AuNCs and 14 days for 98BSA-AuNCs), then (subsequently) slowly decreased—Figure 5, Table S4 (Supporting Information). The first points in Figure 5 were recorded 40 min after the AuNCs syntheses and the last ones, after two months (although the fluorescence signal could be observed much longer, however its intensity substantially decreased). The highest integral fluorescence intensity for all three samples containing different types of BSA was observed on the fourth day. After two months, all the three samples only provided around 30% of the maximal value of fluorescence integral intensity.

Hydrodynamic radius of all three types of BSA-containing samples were re-measured in specific time intervals elapsed from the beginning of the synthesis, 96 h and 168 h, and are presented in Table S5 (Supporting Information). While monomodal size distribution was characteristic for the as-prepared samples, bimodal (for 98BSA-AuNCs) and/or even trimodal (for df98BSA-AuNCs and 96BSA-AuNCs) size distributions were revealed after 96 h and 168 h (with the second and third size distributions around 33–40 nm and over 196 nm, respectively). It points out aggregation of protein and possible further growth of AuNCs.

Generally, it is accepted that several different binding sites on BSA are available for Au (III) and thus, various AuNCs are formed and present within the samples. The AuNCs can be more and/or less accessible to further growth (note: The samples were not dialyzed, thus Au (III) was still present) according to their position within the denaturated BSA structure. If they grow and/or aggregate above a critical size, e.g., until nanoparticles (obviously, even the storage in the fridge does not stop their growth), they lose their fluorescent properties. Therefore, the integral fluorescence intensity varies over time.

The integral emission intensity decreased to half of its maximal value after approximately one month for 98BSA, and earlier in the cases of 96BSA and df98BSA—Figure 5, Table S4 (Supporting Information). This may be related to the presence of FAs in its natural content in 98BSA. As it was revealed, FAs were able to stabilize the structure of BSA to some extent, so that the changes of secondary structure were less pronounced than in 96BSA and df98BSA (Table 1, Figure 1). 

Since we do not possess any experimental technique in our lab that could more precisely determine the exact place of Au and BSA interactions for different types of BSA used, we can only speculate that the accessibility of AuNCs for their further growth is different in 98BSA, df98BSA, and 96BSA. This can explain the observed differences in fluorescence intensity decrease. Fluorescence lifetime values of all three types of AuNCs, prepared using different kinds of BSA precursors and re-measured after three months, are listed in Table 2 (for the sake of a direct comparison with the lifetime values obtained for four-day aged samples). It is evident that the fluorescence decay characteristics were essentially unaltered (i.e., their values repeated within the confidential interval). In other words, the same fluorescent species (possessing long lifetime values) were measured after several months, however their content within each sample was lowered (as can be deduced from the integral fluorescence intensity decrease). This supports the hypothesis of different accessibility of AuNCs within various BSA precursors. The non-monotonic time behavior of integral fluorescence intensity and changes in lifetime values indicate that the absence of fatty acids and/or presence of impurities in the protein play significant roles in AuNCs formation and fluorescence features.

### 3.4. Biological Tolerance of AuNCs

**Figure 6:**
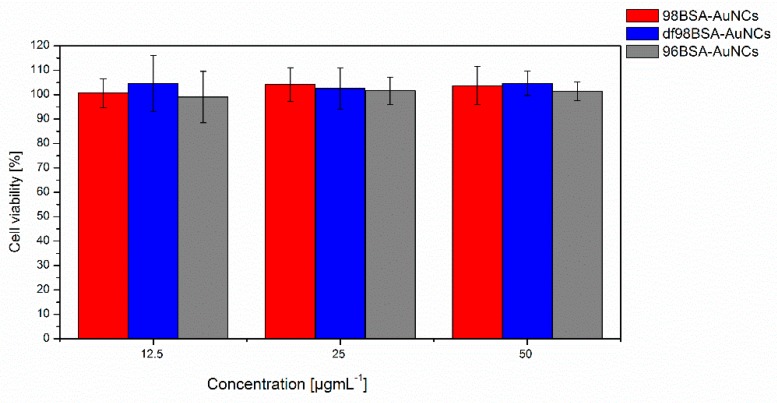
Viability of cells exposed to AuNCs. Value of absorbance was normalized to control cells that were not labeled with AuNCs. Control was set to 100%. Treated cells were labeled for 24 h with 96BSA-AuNCs (grey columns), or 98BSA-AuNCs (red columns), or df98BSA-AuNCs (blue columns) in concentrations of 12.5, 25, or 50 µg Au/mL. Data are expressed as a mean of three independent experiments ± standard deviation (SD).

Testing the biocompatibility, biodegradation, and possible adverse effect of nanomaterials designed for in vivo applications is of high importance because numerous toxic effects of nanoparticles were described [50,51,52]. We performed the cytotoxicity assessment of AuNCs exposure to Hep G2 cell line with Alamar Blue assay. Cells were treated with all three types (98BSA-AuNCs, df98BSA-AuNCs, 96BSA-AuNCs) of NCs (98BSA-AuNCs, df98BSA-AuNCs, 96BSA-AuNCs) for 24 h. As shown in Figure 6, we did not find any negative effect on cell viability in the used concentrations and selected time interval, nor did we find any significant difference between the tested NCs. Cytotoxicity of AuNPs was tested by several other groups. Results vary according to the tested cell line. Patra et al. [53] showed no cytotoxicity effect of gold nanoparticles on viability or cell shape of HepG2 cell line, however the viability of A549 cells decreased rapidly in the presence of the same AuNPs. Gannon et al. [54] did not reveal any negative effect of AuNCs on viability of Panc-1 or Hep3B cell lines. Toxic effect of AuNPs is also highly dependent on nanoparticles´ size, shape, and concentration as documented on various cell lines [52]. Gold nanospheres seem to be less toxic then nanostars or nanorods based on viability or ultrastructural data [55,56].

## 4. Conclusions

It can be concluded that the content of PA and/or other substances in BSA plays a crucial role in the AuNCs formation. The accessibility of cysteine and tyrosine residues is most probably different in 96BSA vs. 98BSA vs. df98BSA, which consequently influences the AuNCs formation and its properties, as it was determined by several spectroscopic and other experimental techniques. In general, the access of auric ions to reactive amino acid residues on protein surface is a critical factor in AuNCs formation. 

Furthermore, the content of PA and/or other substances has a significant impact on AuNCs fluorescent characteristics, such as fluorescence lifetime and quantum yield. The long-term stability followed for several months was investigated and a non-monotonic time behavior of integral fluorescence intensity and negligible changes in lifetime values were revealed. Last, but not least, a very good biological tolerance of AuNCs was evidenced on Hep G2 cell line.

## Figures and Tables

**Table 1:** Calculated α-helix content in albumin either by using BeStSel program (detailed structural changes shown in Table S1 (Supporting information)), or MRE values as in [11,39]. The changes of α-helix content in the course of AuNCs synthetic procedure and data for each type of BSA used in our study are listed.

System	BeStSel Σ Helix [%]	BeStSel Antiparalle+Parallel [%]	BeStSel Turn+Others [%]	Equations (3) and (4) α-helix BSA-Reference [%]	Equations (3) and (4) α-helix BSA-AuNCs [%]
98BSA	47.5	4.8	47.7	52.6	52.6
df98BSA	45.4	6.2	48.4	51.0	51.0
96BSA	45.1	5.1	49.7	51.5	51.5
Influence of HAuCl_4_ or HCl addition					
98BSA+Au^3+^	39.2	14.6	46.2	34.7^a)^	40.1
df98BSA+Au^3+^	36.7	11.8	51.5	34.1^a)^	41.0
96BSA+Au^3+^	37.6	14.9	47.4	32.4^a)^	41.2
Influence of alkalization					
98BSA-Au ^3+^+OH^−^	23.2	23.6	53.2	32.8^a)^	28.2
df98BSA-Au ^3+^+OH^−^	24.8	21.4	53.8	33.2^a)^	26.7
96BSA-Au ^3+^+OH^−^	23.3	22.4	54.3	33.2^a)^	27.0
Influence of microwave irradiation					
98BSA-AuNCs	18.7	23.7	57.5	23.6 ^a)^	19.8
df98BSA-AuNCs	18.1	25	56.9	18.7 ^a)^	19.2
96BSA-AuNCs	17.8	24.4	57.7	21.2 ^a)^	19.3

^a)^ Instead of HAuCl_4_ addition, effect of pH changes caused by HCl and/or NaOH.

**Table 2:** Calculated quantum yield (QY), fluorescence decay components of AuNCs with lifetime values (τ*_i_*) within confidential intervals (*CI*), and corresponding fractional intensities (*f_i_*) measured for the four-day aged AuNCs. The lifetimes were re-measured after three months and the values (τ*_i_, f_i_*) are also listed.

System	QY	τ*_i_* <68% CI>	*f_i_*	τ*_i_*—After 3 Months	
	[%]	[ns]	[%]	[ns]	[%]
98BSA-AuNCs	6.2 ± 0.2	369.2 < 332.7; 423.0>	96.5	395.4 < 356.1; 459.4>	93.8
		11.1 < 7.8; 16.8>	1.3	11.9 < 9.9; 16.2>	1.9
		2.0 < 1.6; 2.5>	1.3	2.7 < 2.3; 3.3>	2.7
		0.2 < 0.1; 0.3>	0.9	0.4 < 0.3; 0.5>	1.6
df98BSA-AuNCs	5.7 ± 0.2	405.5 < 353.0; 479.8>	96.8	371.4 < 342.1; 436.0>	94.4
		14.2 < 9.9; 20.3>	1.2	11.7 < 9.0; 14.3>	2.0
		2.3 < 1.6; 2.9>	1.2	2.2 < 1.6; 3.0>	2.2
		0.3 < 0.2;0.5>	0.8	0.4 < 0.3; 0.5>	1.4
96BSA-AuNCs	4.3 ± 0.2	452.0 < 371.2; 520.6>	97.7	427.2 < 352.5; 456.0>	95.4
		14.4 < 10.7; 18.4>	0.9	11.6 < 9.8; 13.6>	1.6
		2.3 < 1.7; 2.7>	0.8	2.5 < 2.0; 2.9>	1.9
		0.2 < 0.1; 0.3>	0.6	0.4 < 0.3; 0.4>	1.1

**Table 3:** Calculated quantum yield (QY), hydrodynamic radius, and zeta potential values of the samples where PA was added to df98BSA at various molar ratios (PA:BSA being 1:1, 2:1, and 6:1) prior to or after AuNCs synthesis.

System	QY [%]	DLS [nm]	Zeta Potential [mV]
PA:df98BSA(1:1)-AuNCs	6.2 ± 0.1	12.6 ± 3.2	−17.8 ± 1.5
PA:df98BSA(2:1)-AuNCs	6.6 ± 0.4	12.8 ± 1.8	−18.1 ± 1.2
PA:df98BSA(6:1)-AuNCs	7.0 ± 0.1	14.3 ± 3.2	−26.3 ± 3.6
df98BSA-AuNCs:PA (1:6)	7.2 ± 0.3	11.8 ± 1.7	−18.0 ± 1.7

## Supplementary Materials

The following are available online at https://www.mdpi.com/2079-4991/10/2/343/s1. Figure S1: CD spectra for key steps of the final AuNCs syntheses, Table S1: Detailed changes in secondary structure determined by BeStSel algorithm, Table S2: Fraction of secondary structure elements of selected samples derived from IR spectra, Table S3: Hydrodynamic diameters of the initial proteins used in this study and their successive changes in the course of AuNCs formation determined by DLS, Figure S2: UV/Vis absorption and fluorescence intensity of 98BSA-AuNCs, Figure S3: Fluorescence intensity of 98BSA-AuNCs, df98BSA-AuNCs and 96BSA-AuNCs, Figure S4: Lifetime measurement with fit and distribution of residuals, Figure S5: IR spectra of the final systems containing various PA:BSA molar ratios, Figure S6: 3D excitation-emission maps of df98BSA-AuNCs, PA:df98BSA-AuNCs(6:1) and df98BSA-AuNCs:PA(1:6), Figure S7: 96BSA-AuNCs: CD spectra, fluorescence excitation-emission 3D maps and IR spectra, Table S4: Changes of integral fluorescence intensity values of 96BSA-AuNCs, 98BSA-AuNCs, and df98BSA-AuNCs as a function of time, Table S5: Time evolution of hydrodynamic radius of various BSA-AuNCs.
