# Supplementary material for: The Effect of Fatty Acids and BSA Purity on Synthesis and Properties of Fluorescent Gold Nanoclusters
Source: Nanomaterials (Basel). 2020 Feb 17;10(2):343. doi: 10.3390/nano10020343 (PMC7075172; doi:10.3390/nano10020343)
Supplement: Supplementary file 1 [file nanomaterials-10-00343-s001.pdf]

nanomaterials

Supporting Information

The Effect of Fatty Acids and BSA Purity on Synthesis and Properties of Fluorescent Gold Nanoclusters

Pavlína Andrýsková, Karolína Machalová Šišková *, Šárka Michetschlägerová, Klára Jiráková, Martin Kubala and Daniel Jirák

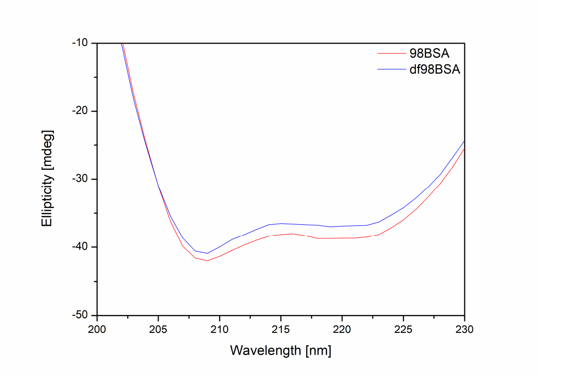

(**A**)

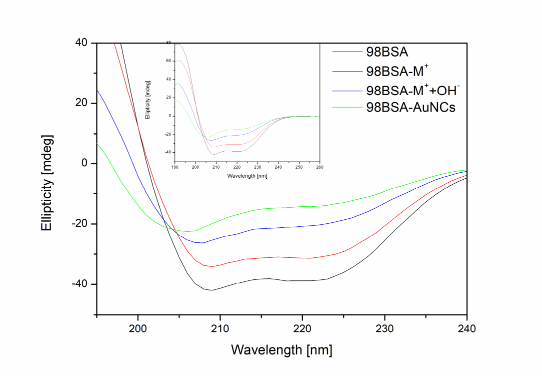

(**B**)

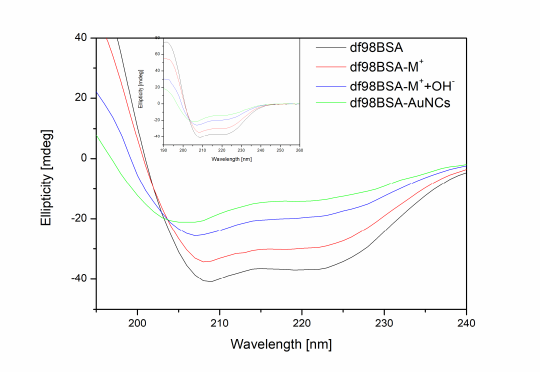

(**C**)

Figure S1. CD spectra for key steps of the final AuNCs syntheses. (**A**) Comparison of CD spectra of pure 98BSA and df98BSA dissolved in deionized water. (**B**) CD spectra of pure 98BSA (black line), after the addition of $HAuCl_4$ (red line), after alkaline pH adjustment (blue line) and the final AuNCs, i.e. after MW treatment (green line). (**C**) Similarly as in (**B**), for the case of df98BSA as a starting solution.

Table S1. Detailed changes in secondary structure of the initial proteins used in this study and their successive changes in the course of AuNCs formation determined by BeStSel algorithm.

	**Helix 1 Regular α-helix [%]**	**Helix 2 distorted α-helix [%]**	**Anti 1 Left-twisted β-strand [%]**	**Anti 2 Relaxed β-strand [%]**	**Anti 3 Right-twisted β-strand [%]**	**Parallel β-strand [%]**	**Turn [%]**	**Others [%]**
98BSA	31.3	16.2	1.6	1.6	0	1.6	11.5	36.2
df98BSA	30.1	15.3	1.1	2	1.2	1.9	12.1	36.3
96BSA	29.6	15.5	1.3	1.4	0	2.4	12.1	37.6
Influence of $HAuCl_4$								
$98BSA+Au^{3+}$	25.5	13.7	2.2	3.5	5.7	3.2	12	34.2
$df98BSA+Au^{3+}$	22.9	13.8	1.4	3.1	4.1	3.2	12.6	38.9
$96BSA+Au^{3+}$	24.2	13.4	1.9	3.8	6.9	2.3	12	35.4
Influence of alkalization								
$98BSA-Au^{3+}+OH^{-}$	13.9	9.3	0.5	6.8	12.5	3.8	13.3	39.9
$df98BSA-Au^{3+}+OH^{-}$	14.5	10.3	1.2	6.3	12	1.9	13.2	40.6
$96BSA-Au^{3+}+OH^{-}$	13.6	9.7	0.9	6.5	12.4	2.6	13.1	41.2
Influence of microwave irradiation								
98BSA-AuNCs	10.2	8.5	0.1	8	15.6	0	14.6	42.9
df98BSA-AuNCs	9.9	8.2	0.3	8.8	15.9	0	14.5	42.4
96BSA-AuNCs	9.5	8.3	0	8.6	15.8	0	14.8	42.9

Table S2. Fraction of secondary structure elements of selected samples derived from IR spectra and determined after amide I band deconvolution into four gaussian peaks. The area percentage of the gaussian curves and peak assignment (according to the ref. Zhang et al., J. Phys. Chem. C 2013) are listed.

**Peak position ±4 cm^{-1}**	**Assignment**	**Area percentage for sample Ref-98BSA**	**Area percentage for sample 98BSA-AuNCs**	**Area percentage for sample Ref-df98BSA**	**Area percentage for sample df98BSA-AuNCs**
[cm^{-1}]		[%]	[%]	[%]	[%]
1654	Buried alpha helix	12	10	12	5
1634	Exposed alpha helix	38	24	40	29
1643	Random coil	22	42	24	45
1676	Turn structures	28	24	24	21

Table S3. Hydrodynamic diameters of the initial proteins used in this study and their successive changes in the course of AuNCs formation determined by DLS. Δ1 and Δ2 are calculated by following subtractions: Δ1 = (BSA-Au^{3+} + OH^-) − (BSA) and Δ2 = (BSA-AuNCs) − (BSA-Au^{3+} + OH^-). Zeta potential values determined for the final AuNCs are also listed.

	**DLS BSA [nm]**	**DLS BSA+Au^{3+} [nm]**	**DLS BSA-Au^{3+} + OH^- [nm]**	**DLS BSA-AuNCs [nm]**	**Δ1 [nm]**	**Δ2 [nm]**	**Zeta potential BSA-AuNCs [mV]**
98BSA-AuNCs	8.6 ± 0.5	8.1 ± 0.3	10.0 ± 1.7	13.6 ± 3.5	2.4	3.6	−16.0 ± 0.7
df98BSA-AuNCs	6.4 ± 0.2	7.8 ± 2.3	13.2 ± 1.4	14.4 ± 2.0	6.8	1.2	−14.5 ± 1.6
96BSA-AuNCs	5.4 ± 0.3	5.8 ± 0.1	9.2 ± 1.5	10.5 ± 1.5	3.8	1.3	−18.5 ± 0.3

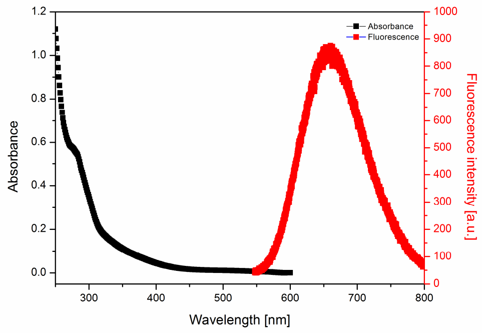

Figure S2. UV/Vis absorption (black line) and fluorescence intensity (red line) of 98BSA-AuNCs.

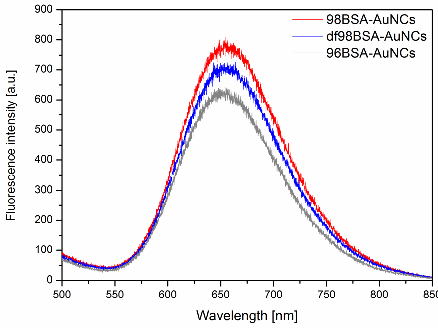

Figure S3. Fluorescence intensity of 98BSA-AuNCs (red line), df98BSA-AuNCs (blue line) and 96BSA-AuNCs (grey line).

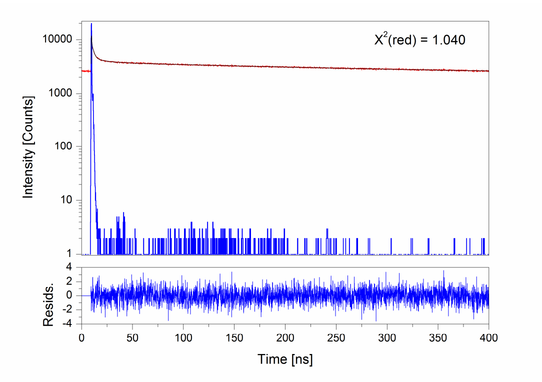

(**A**)

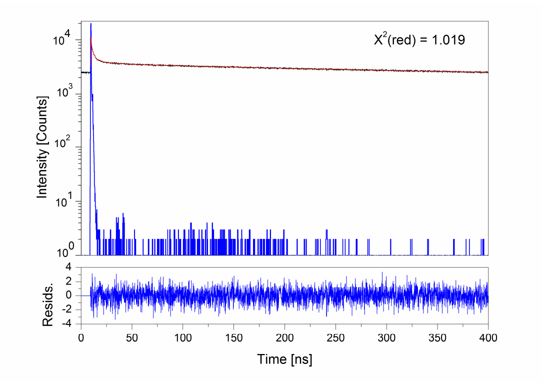

(**B**)

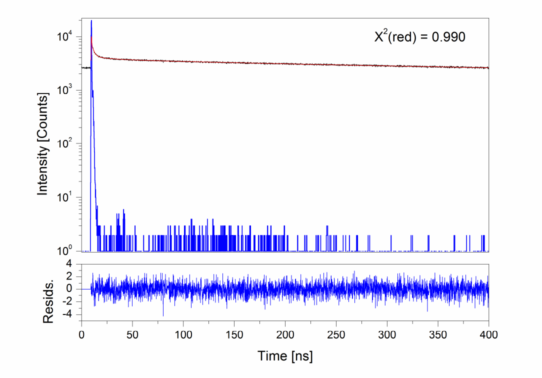

(**C**)

Figure S4. Lifetime measurement (black line) with fit (red line) and distribution of residuals (blue line) of 98BSA-AuNCs (**A**), df98BSA-AuNCs (**B**) and 96BSA-AuNCs (**C**).

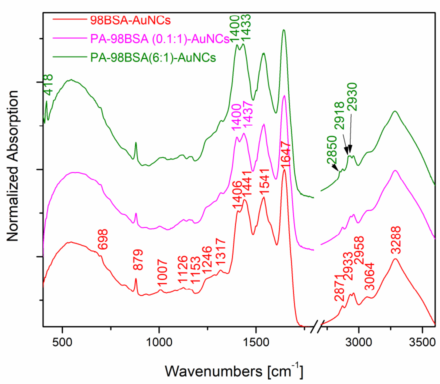

(**A**)

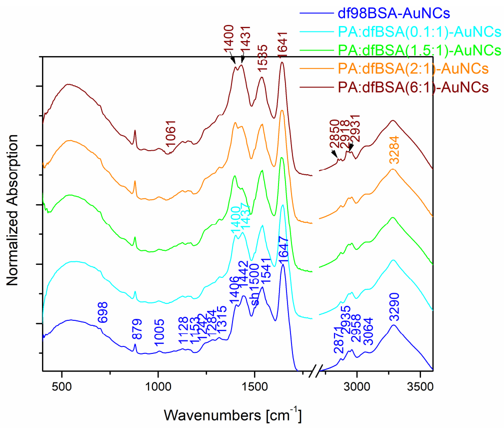

(**B**)

Figure S5. IR spectra of the final systems containing various PA:BSA molar ratios which were used for AuNCs syntheses: (**A**) 98BSA-AuNCs, (**B**) df98BSA-AuNCs.

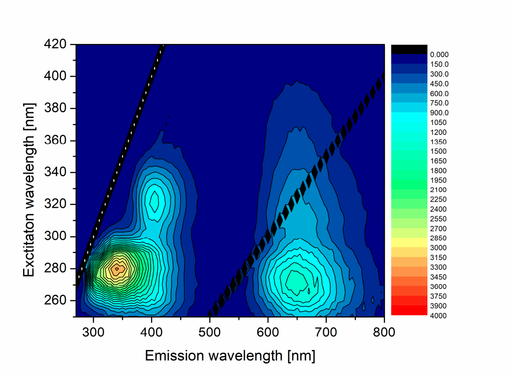

(**A**)

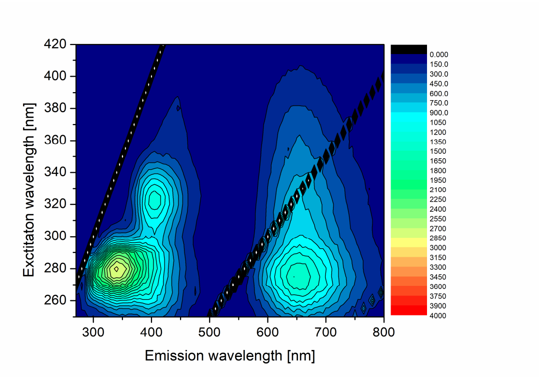

(**B**)

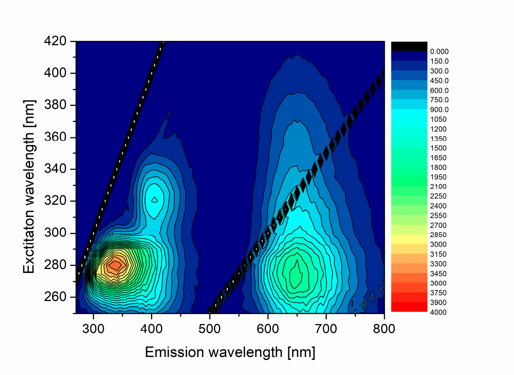

(**C**)

Figure S6. 3D excitation-emission maps of (**A**) df98BSA-AuNCs (**B**) PA:df98BSA-AuNCs(6:1) and (**C**) df98BSA-AuNCs:PA(1:6).

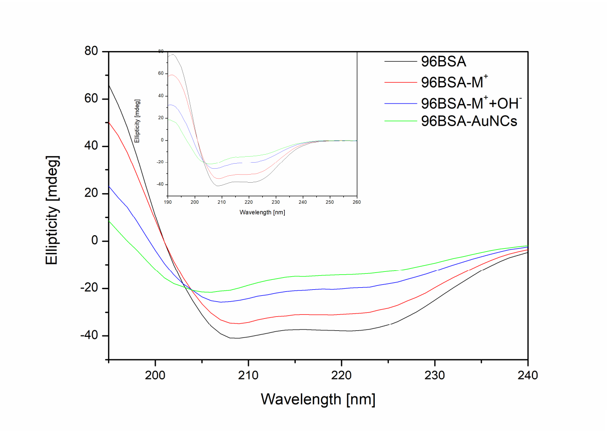

(**A**)

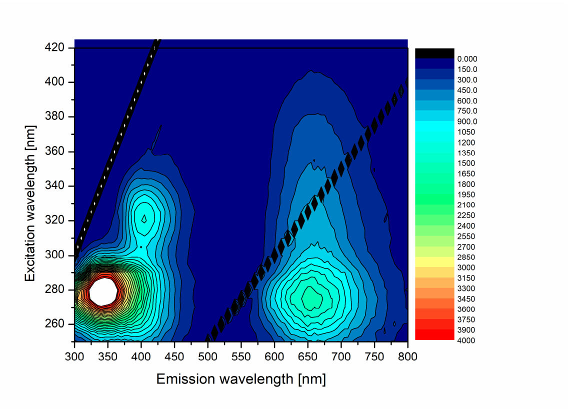

(**B**)

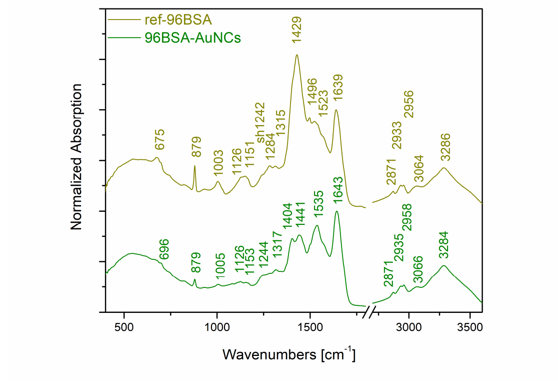

(**C**)

Figure S7. 96BSA-AuNCs: (**A**) CD spectra**,** (**B**) fluorescence excitation-emission 3D maps, (**C**) IR spectra (as a reference sample 96BSA treated in the same way as 96BSA-AuNCs is employed).

Table S4. Changes of integral fluorescence intensity values of 96BSA-AuNCs, 98BSA-AuNCs, and df98BSA-AuNCs (determined in the emission range 550–800 nm, using 280 nm excitation) as a function of time (expressed in hours).

	Time [h]								
	**0**	**21**	**24**	**48**	**96**	**168**	**336**	**720**	**1440**
98BSA-AuNCs	0.53	0.86	0.91	0.86	1.00	0.98	0.57	0.51	0.36
df98BSA-AuNCs	0.62	0.75	0.88	0.83	1.00	0.74	0.51	0.40	0.29
96BSA-AuNCs	0.53	0.87	0.82	0.87	1.00	0.59	0.62	0.44	0.32

Table S5. Time evolution of hydrodynamic radius of various BSA-AuNCs.

**System**	**BSA-AuNCs 96 h**		**BSA-AuNCs 168 h**	
	[nm]	[%]	[nm]	[%]
98BSA-AuNCs	8.2 ± 2.0<br>33.8 ± 2.4	10.8<br>89.2	11.4 ± 2.5<br>32.7 ± 4.0<br>-----	15.4<br>84.6
df98BSA-AuNCs	11.5 ± 1.7<br>40.8 ± 6.2	19.2<br>80.8	10.5 ± 2.9<br>42.3 ± 4.7<br>196.9 ± 50.3	17.8<br>42.5<br>39.7
96BSA-AuNCs	12.4 ± 2.0<br>33.3 ± 3.9<br>270.6 ± 69.1	26.4<br>66.3<br>7.3	11.3 ± 1.3<br>33.1 ± 3.8<br>304.8 ± 42.6<br>620.3 ± 45.3	15.2<br>68.3<br>6.5<br>10.0
